# Silicone Oil Opacification after Prolonged Intraocular Retention

**DOI:** 10.18502/jovr.v16i2.9096

**Published:** 2021-04-29

**Authors:** Parijat Chandra, Vinod Kumar, Brijesh Takkar, Devesh Kumawat

**Affiliations:** ^1^Dr. Rajendra Prasad Centre for Ophthalmic Sciences, All India Institute of Medical Sciences, New Delhi, India; ^2^Department of Ophthalmology, All India Institute of Medical Sciences, Bhopal, India

##  PRESENTATION 

A 13-year-old boy had closed globe injury on the right eye with a cricket ball two years back and presented with loss of vision for the past one year. The right eye had perception of light, intraocular pressure of 32 mmHg, total cataractous lens, 210 degrees angle recession, and retinal detachment on B scan ultrasonography. The left eye was normal.

The right eye underwent pars plana lensectomy and vitrectomy with 1000cs silicone oil tamponade. The neuroretinal rim was found to be pale with cup/disc ratio of 0.5. He was prescribed routine postoperative care. At one week, the best-corrected visual acuity (BCVA) was 20/400, media was clear, and retina was attached. The intraocular pressure was 20 mmHg with topical timolol maleate (0.5%) BID and brimonidine tartrate (0.15%) BID. He was scheduled to follow-up after three weeks but did not show up until one year later when he presented with gradual worsening of vision in the right eye. During this period, he did not use any medications. BCVA of the right eye was perception of light with inaccurate projection. The intraocular pressure was 48 mmHg. The silicone oil had been opacified with no evidence of emulsification. There was no fundal glow through the opaque silicone oil. However, the peripheral retina around the oil meniscus was clearly visible and attached.

**Figure 1:**
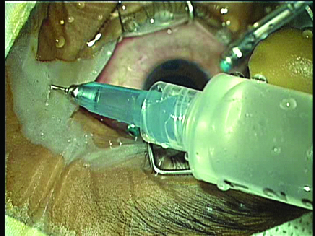
Opaque silicone oil intermixed with infusion fluid in extraction syringe intraoperatively.

**Figure 2:**
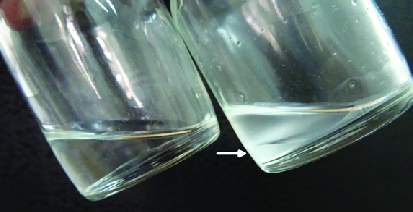
Left bottle: Unused clear silicone oil. Right bottle: Opaque silicone oil with infusion fluid at top. White opaque oil is settled at the bottom (white arrow).

The child underwent uneventful silicone oil removal. The retina was well attached, but a total loss of the disc neural rim was noted. The aspirated silicone oil was opaque and white in color [Figure 1], and heavier than normal silicone oil as it settled to the bottom under the infusion fluid [Figure 2]. On first monthly follow-up, the BCVA was perception of light with accurate projection, retina was attached, and intraocular pressure was 18 mmHg with topical medications.

##  DISCUSSION

Silicone oil emulsification is a common complication, observed in cases where long-standing tamponade is performed following vitreoretinal surgery.^[1]^ In a conference proceedings, Spitzer et al reported opacification of 5000cs silicone oil without emulsification in 12 cases occurring a few weeks after surgery, traced to a single production lot, wherein the physicochemical analyses had revealed that the opaque oil was more heat-stable and was possibly deficient of a stabilizing agent against coloration.^[2]^

Ciardella et al previously reported that retained perfluorocarbon liquid bubbles in the silicone oil-filled vitreous cavity might lead to an opaque fluid consisting of both silicone oil and micro-dispersed perfluorocarbon liquid.^[3]^ It is notable that no surgical adjuncts (intraocular steroids, perfluorocarbon liquids, or dyes) was used during the first surgery in the current case. It is unclear what complex physio–chemical changes occurred in the oil that may have led to the opacification.

Thus, long-term retained silicone oil can rarely lose its transparency in the absence of emulsification. However, the situation may be disturbing for the patient due to significant loss of visual acuity. This case highlights the need for timely silicone oil removal.

##  Financial Support and Sponsorship

Nil.

##  Conflicts of Interest

There are no conflicts of interest.
